# Sequential Treatment with Activin and Hepatocyte Growth Factor Induces FOXM1 to Promote Colorectal Cancer Liver Metastasis

**DOI:** 10.1155/2022/8996203

**Published:** 2022-12-23

**Authors:** Hong Peng, Ting Ye, Lei Deng, Xiaofang Yang, Zheng Jiang, Jinjun Guo

**Affiliations:** ^1^Bishan Hospital of Chongqing, Bishan Hospital of Chongqing Medical University, Chongqing, China; ^2^Chongqing Emergency Medical Center, Chongqing University Central Hospital, School of Medicine, Chongqing University, Chongqing, China; ^3^Department of Gastroenterology, The First Affiliated Hospital of Chongqing Medical University, Chongqing, China

## Abstract

**Background:**

Cancer stem cells (CSCs) are involved in liver metastasis in colorectal cancer (CRC). Activin and hepatocyte growth factor (HGF) are important regulators of stem cell properties. This study was performed to explore the effect of activin and HGF on CRC invasion and metastasis. The key genes involved in the action of activin and HGF in CRC were identified.

**Methods:**

HCT116 CRC cells were sequentially treated with activin and HGF and examined for migration and invasion in vitro and liver metastasis in vivo. RNA sequencing was performed to identify differentially expressed genes in response to activin and HGF.

**Results:**

Sequential treatment with activin and HGF-enhanced CRC cell migration, invasion, and metastasis. CXCR4 and AFP expressions were increased by activin and HGF treatment. Knockdown of FOXM1 blocked liver metastasis from HCT116 cells pretreated with activin and HGF and suppressed CXCR4 and AFP expression. Activin alone increased the mRNA and protein expression of FOXM1. In contrast, HGF alone enhanced the phosphorylation of FOXM1, without altering the total protein level of FOXM1. SMAD2 was required for activin-mediated FOXM1 induction. FOXM1 transactivated CXCR4 by directly binding to the promoter of *CXCR4*. Additionally, CXCR4 regulated AFP expression through the NF-*κ*B pathway.

**Conclusions:**

Sequential treatment with activin and HGF accelerates CRC invasion and liver metastasis, which involves the upregulation and activation of FOXM1 and induction of CXCR4 and AFP.

## 1. Introduction

Colorectal cancer (CRC) is the third most common cancer globally and the second leading cause of cancer-related deaths [1]. CRC has an extremely high propensity to spread to the liver, consequently leading to a poor prognosis [2]. Most CRC patients with liver metastasis have lost the opportunity to receive curative resection and die of the disease within 5 years [2]. Hence, it is significant to develop therapeutic strategies that can prevent liver metastasis in CRC patients [3]. However, the mechanism underlying CRC liver metastasis remains unclear.

Cancer stem cells (CSCs), which represent a small subset of tumor cells (1–10%), are responsible for tumor growth, relapse, and treatment resistance [4, 5]. CSCs have been shown to be involved in CRC liver metastasis [5]. Activin and hepatocyte growth factor (HGF) have the capacity of inducing liver progenitor cells from human embryonic stem cells [6]. Activin can promote stem cells to differentiate into definitive endoderm cells expressing characteristic markers, such as CXCR4, FOXA2, SOX17, and CER [7]. In turn, HGF induces the differentiation of definitive endoderm cells into liver progenitor cells, which are characterized by alpha-fetoprotein (AFP) expression [7]. Recent studies have suggested that activin is overexpressed in stage IV CRC [8] and plays an essential role in CRC plasticity and metastasis [9]. Moreover, the activin pathway enhances CRC stem cell self-renewal and tumor progression [10]. Autocrine HGF/c-Met activation can induce common adenocarcinoma cells to dedifferentiate to a CSC state [11]. Given the activities of activin and HGF in regulating CSC, we hypothesized that activin and HGF might have an important impact on CRC metastasis through the modulation of cancer stemness-related factors.

To test the hypothesis, this study was conducted to determine the effect of activin and HGF on CRC invasion and metastasis. Transcriptome analysis was performed to identify key genes involved in the action of activin and HGF in CRC.

## 2. Methods

### 2.1. Patients and Tissue Samples

Paraffin-embedded primary tumor and paired liver metastasis samples were obtained from CRC patients with liver metastasis who were treated at Bishan Hospital of Chongqing (Chongqing, China). The samples were analyzed using immunohistochemical staining with CXCR4 and AFP primary antibodies (Abcam, Cambridge, MA, USA) and an Alexa Fluor-conjugated secondary antibody (1 : 500; Molecular Probes, Eugene, OR, USA). The Medical Ethics Committee of Bishan Hospital of Chongqing approved the study protocol. The patients provided informed consent and had not received radiation or chemotherapy before surgery.

### 2.2. Cell Culture and Transfection

Human CRC cell line HCT116 was obtained from the American Tissue Culture Collection (Manassas, VA, USA) and cultured under the recommended conditions. According to the manufacturer's protocol, transfections were performed in 70–80% confluent cells using Lipofectamine 3000 (Invitrogen, Carlsbad, CA, USA).

### 2.3. Activin and HGF Treatment

HCT116 cells were treated sequentially with activin and HGF, as reported previously [12]. Briefly, at 70% confluence, HCT116 cells were exposed to 100 ng/mL activin A (Peprotech, Rocky Hill, NJ, USA) for 5 days. The cells were then treated with 20 ng/mL HGF (Peprotech) for another 5 days. Afterward, the cells were tested for gene expression, migration, and invasion.

### 2.4. Plasmids and Short Hairpin RNAs (shRNAs)

The FOXM1-and CXCL12-expressing plasmids were constructed by cloning corresponding full-length cDNAs into the pcDNA3.1/HA or GFP vector (Invitrogen). Lentiviral vectors containing FOXM1 shRNA (5′-CGCTACTTGACATTGGACCAA-3′) and negative control were obtained from Tsingke (Wuhan, China). 

### 2.5. RNA Extraction and Real-Time PCR Analysis

Total RNA was isolated using RNAiso Plus (Takara, Shiga, Japan), and 1 *μ*g RNA was used to synthesize cDNA with the PrimeScript™RT reagent Kit with gDNA Eraser (Takara). SYBR Green master mix (Bio-Rad, Hercules, CA, USA) was used for real-time PCR. *β*-Actin was used as an internal control. The primers used for real-time PCR are listed in Table 1.

### 2.6. Western Blotting Analysis

The total protein lysate of cells was prepared in radioimmunoprecipitation assay buffer containing 1 mM phenylmethylsulfonyl fluoride (Beyotime, Shanghai, China). A bicinchoninic acid protein assay kit (Beyotime) was used to determine the protein concentrations. Equal amounts (20 *μ*g) of proteins were separated using sodium dodecyl sulfate-polyacrylamide gel electrophoresis and transferred onto a polyvinylidene difluoride membrane (Millipore, Billerica, MA, USA). Subsequently, the membrane was incubated in 5% nonfat milk in Tris-buffered saline (TBS) for 1 h at room temperature. The following primary antibodies were incubated with the membrane at 4°C overnight: anti-CXCR4 (cat:#ab181020, 1 : 1,000) from Abcam (Cambridge, MA, USA), anti-AFP (cat:#4448, 1 : 1,000), anti-FOXM1 (cat:#20459, 1 : 1,000), phosphor-FOXM1 (cat:#14170, 1 : 1,000), and anti-p65 (cat:#3033, 1 : 1,000) from Cell Signaling Technology (Danvers, MA, USA), as well as anti-*β*-actin (cat:#66009-1-Ig, 1 : 5,000) from Proteintech. After washing with 0.5% Tween-20 in TBS, the membrane was incubated with horseradish peroxidase-conjugated secondary antibody (Absin, Shanghai, China) for 1 h at room temperature. Protein signals were developed with enhanced chemiluminescence.

### 2.7. Transwell Migration and Invasion Assays

Cell migration was tested using the 24-well transwell with polycarbonate filters. Activin- and HGF-treated cells were suspended in a serum-free medium and added to the upper chamber at a density of 6 × 10^3^ cells/well. Dulbecco's modified Eagle medium containing 10% fetal bovine serum was added to the lower chamber. After incubation for 24 h, the cells were fixed with 4% formaldehyde and stained with crystal violet for 5 min. Cell invasion was assayed using a similar method. Matrigel (BD Biosciences, San Jose, CA, USA) was added to each well and incubated for 5 h at 37°C before the cells were seeded to the upper chamber of the transwell. The cells that invaded through the Matrigel were counted after culturing for 48 h. The average number of migrating or invading cells was calculated in five fields (200x) on each membrane.

### 2.8. In vitro Wound-Healing Assay

The wound-healing assay was performed using a 12-well cell culture plate. A 200-*μ*L sterile pipette tip was used to make a scratch in a cell layer of approximately 100% confluence. After incubation for 48 h in a serum-free medium, the rate of wound closure was measured.

### 2.9. RNA-Sequencing (RNA-Seq) Analysis

Next-generation sequencing libraries were constructed by depleting ribosomal RNA using the Human/Mouse/Rat Ribo-Zero rRNA Removal Kit for Illumina (Epicenter, Madison, WI, USA). At elevated temperatures, the poly(A)+ or poly(A)− RNA fractions were cleaved into small fragments by divalent cations to ensure purification. A cDNA library was constructed by reverse transcription of the cleaved RNA fragments. The average insert size for the paired-end libraries was 250–350 base pairs. The cDNA libraries were sequenced on an Illumina NovaSeq™ 6000 system (LC-Bio Technology CO., Ltd., Hangzhou, China) following the recommended protocol. The gene expression data have been deposited in the Gene Expression Omnibus (GEO) database under accession number GSE205986.

### 2.10. Animal Studies

Animal experiments were performed according to the NIH Guide for the Care and Use of Laboratory Animals. The protocol was approved by the Institutional Animal Care and Use Committee of Chongqing Medical University. Five-week-old female nude (nu/nu) mice were purchased from GemPharmatech Co., Ltd. (Nanjing, China). HCT116 cells with indicated treatments (5 × 10^5^ cells/mouse) were injected into the spleen of the nude mice using a 29-gauge needle. Animals were monitored daily for adverse effects. Each group contained 10 nude mice. Six weeks after cell injection, the mice were sacrificed. An autopsy was performed to check for tumor metastasis. Metastatic lesions in the liver were counted.

### 2.11. Luciferase Reporter Assay

The *CXCR4* promoter (−1995 to +2 bp) and its truncated fragments (−912 to +2 bp, −613 to +2 bp, and −421 to +2 bp) were cloned into a pGL3-Basic vector. All constructs were verified by sequencing. For the luciferase reporter assay, cells were plated in 24-well plates and cotransfected with luciferase reporter constructs, FOXM1-expressing plasmid, and pRL-TK Renilla luciferase control reporter vectors. Luciferase activity was measured 36 h after cell transfection using the dual-luciferase reporter assay system (Promega, Madison, WI, USA) according to the manufacturer's instructions.

### 2.12. Chromatin Immunoprecipitation (ChIP) Assay

Cells were fixed with 1% formaldehyde for 10 min at room temperature. Sonicated cell lysates were subjected to immunoprecipitation using 5 *μ*g anti-FOXM1 (Cell Signaling Technology) or isotype control IgG. Immunoprecipitated DNA was extracted and subjected to PCR analysis. Primers used for ChIP are listed in Table 1.

### 2.13. Statistical Analysis

All statistical analyses were performed using SPSS software (version 22.0, SPSS, Inc., Chicago, IL, USA). Differences between the two groups were determined by Student's *t*-test. Differences among groups were assessed using a one-way analysis of variance. Data are expressed as the means ± standard deviation. *P*  <  0.05 was considered statistically significant.

## 3. Results

### 3.1. Sequential Treatment with Activin and HGF Promotes CRC Invasion and Metastasis

**Figure 1:**
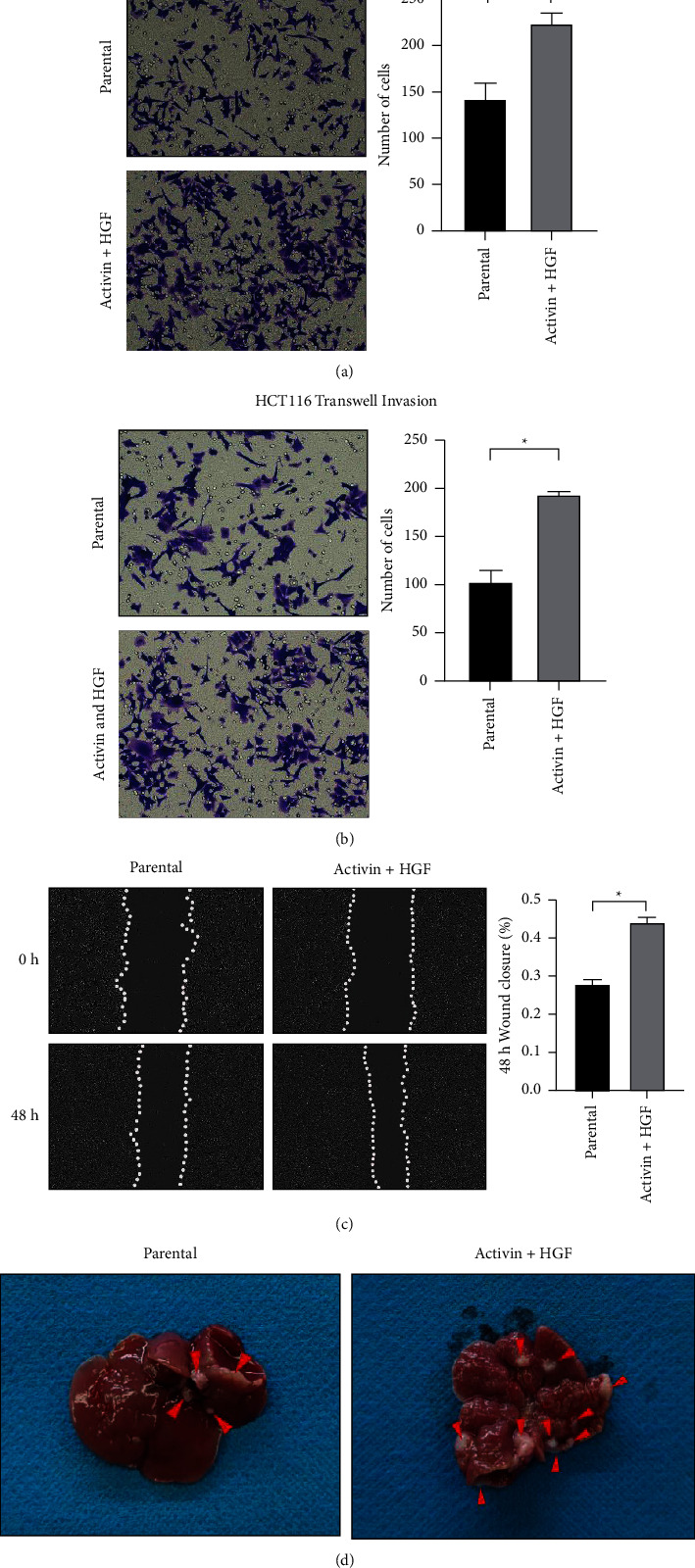
Sequential treatment with activin and hepatocyte growth factor (HGF) promotes cell migration and invasion in vitro and liver metastases in vivo. (a–b) Transwell assays demonstrate that sequential treatment with activin and HGF increases the migration and invasion abilities of HCT116 cells. (c) Wound-healing assays show that sequential treatment with activin and HGF enhances the wound-healing capacity of HCT116 cells. (d) Sequential treatment with activin and HGF promotes liver metastases in nude mice. ^*∗*^*P* < 0.05.

To investigate the effect of sequential treatment with activin and HGF on CRC biology, HCT116 CRC cells were treated initially with activin for 5 days, followed by HGF for another 5 days. Transwell assays revealed that activin and HGF treatment increased the migration and invasion abilities of HCT116 cells (Figures 1(a) and 1(b)). In vitro wound-healing assays demonstrated that the motility of HCT116 cells was enhanced in response to activin and HGF treatment (Figure 1(c)). To determine whether activin and HGF treatment had a long-term impact on CRC cells, HCT116 cells were sequentially treated with activin and HGF and then injected into the spleen of nude mice. As shown in Figure 1(d), activin and HGF treatment of HCT116 cells facilitated the development of metastatic lesions in the liver. These data indicate the prometastatic effect of activin and HGF on CRC cells.

### 3.2. Activin and HGF Treatment Induces CXCR4 and AFP Expression in CRC Cells

**Figure 2:**
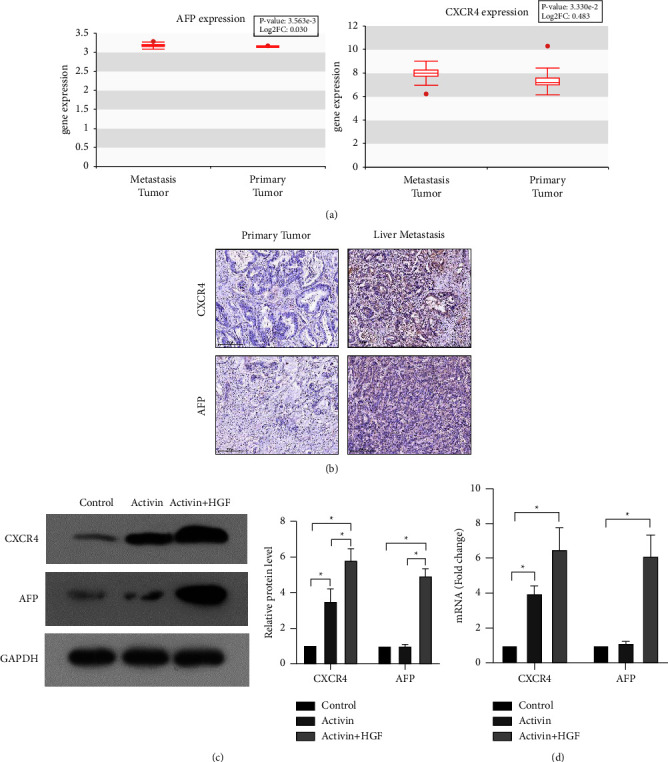
Activin and HGF treatment induces CXCR4 and AFP expression in CRC cells. (a) Analysis of data from the human cancer metastasis database (http://hcmdb.i-sanger.com/index) reveals that CXCR4 and AFP are expressed at higher levels in liver metastases than in primary colon tumors. (b) Immunohistochemical analysis of CXCR4 and AFP levels in liver metastases and primary tumors. (c-d) The effect of activin and HGF treatment on the expression of CXCR4 and AFP in CRC cells.  ^*∗*^*P* < 0.05.

CXCR4 and AFP are recognized as biomarkers of endoderm cells and liver progenitor cells, respectively. Analysis of data from the Human Cancer Metastasis Database (http://hcmdb.i-sanger.com/index) revealed that CXCR4 and AFP were expressed at higher levels in liver metastases than in primary colon tumors (Figure 2(a)). Immunohistochemical analysis confirmed that CXCR4 and AFP levels were increased in liver metastases compared to primary colon tumors (Figure 2(b)). Next, we checked the effect of activin and HGF treatment on the expression of CXCR4 and AFP in CRC cells. Activin treatment alone significantly upregulated the expression of CXCR4 but not AFP in HCT116 cells (Figures 2(c) and 2(d)). Sequential treatment with activin and HGF led to a significant increase in the expression of both CXCR4 and AFP in HCT116 cells (Figures 2(c) and 2(d)).

### 3.3. FOXM1 is Involved in the Prometastatic Activity of Activin and HGF in CRC

**Figure 3:**
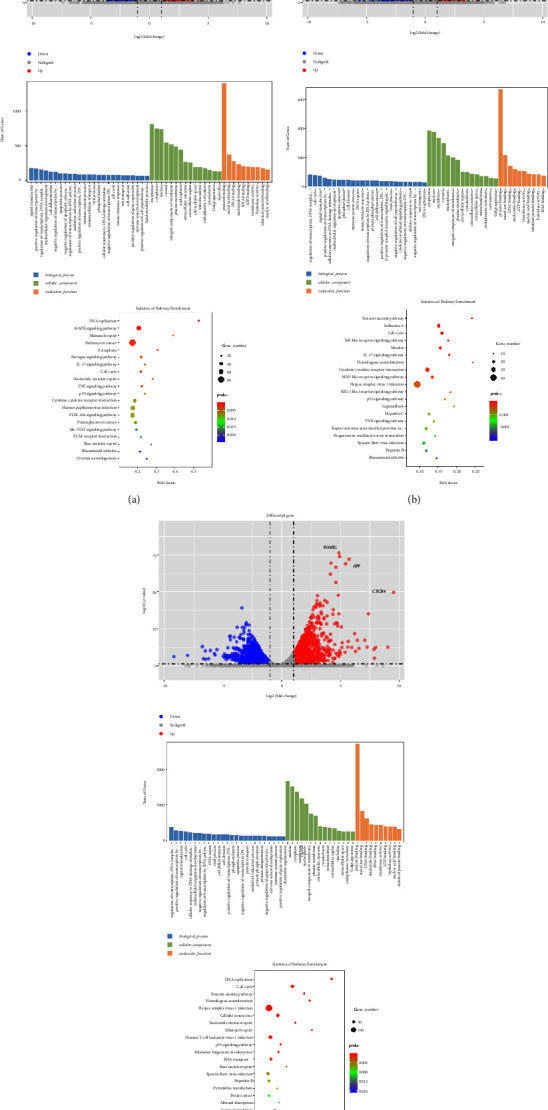
Transcriptome sequencing performed in HCT116 cells subjected to activin alone or in combination with HGF treatment: (a) activin *vs.* parental; (b) activin and HGF *vs.* activin; (c) activin and HGF *vs.* parental.

To explore the mechanism underlying the prometastatic effect of activin and HGF on CRC, transcriptome sequencing was performed in HCT116 cells subjected to activin alone or in combination with HGF treatment. RNA-Seq analysis revealed that the gene expression profiles significantly differed in response to activin and HGF treatment (Figure 3). A total of 2,590 genes were differentially expressed between the control and activin-treated cells and 1,302 genes between the activin-treated and activin + HGF-treated cells (Figures 3(a) and 3(b)). In particular, FOXM1, CXCR4, and AFP were induced by sequential activin and HGF treatment (Figure 3(c)). Gene Ontology analysis revealed that the differentially expressed genes in response to activin and HGF treatment were enriched in several critical cancer-related processes, including “pathways in cancer,” “cell differentiation,” and “signal transduction.”

**Figure 4:**
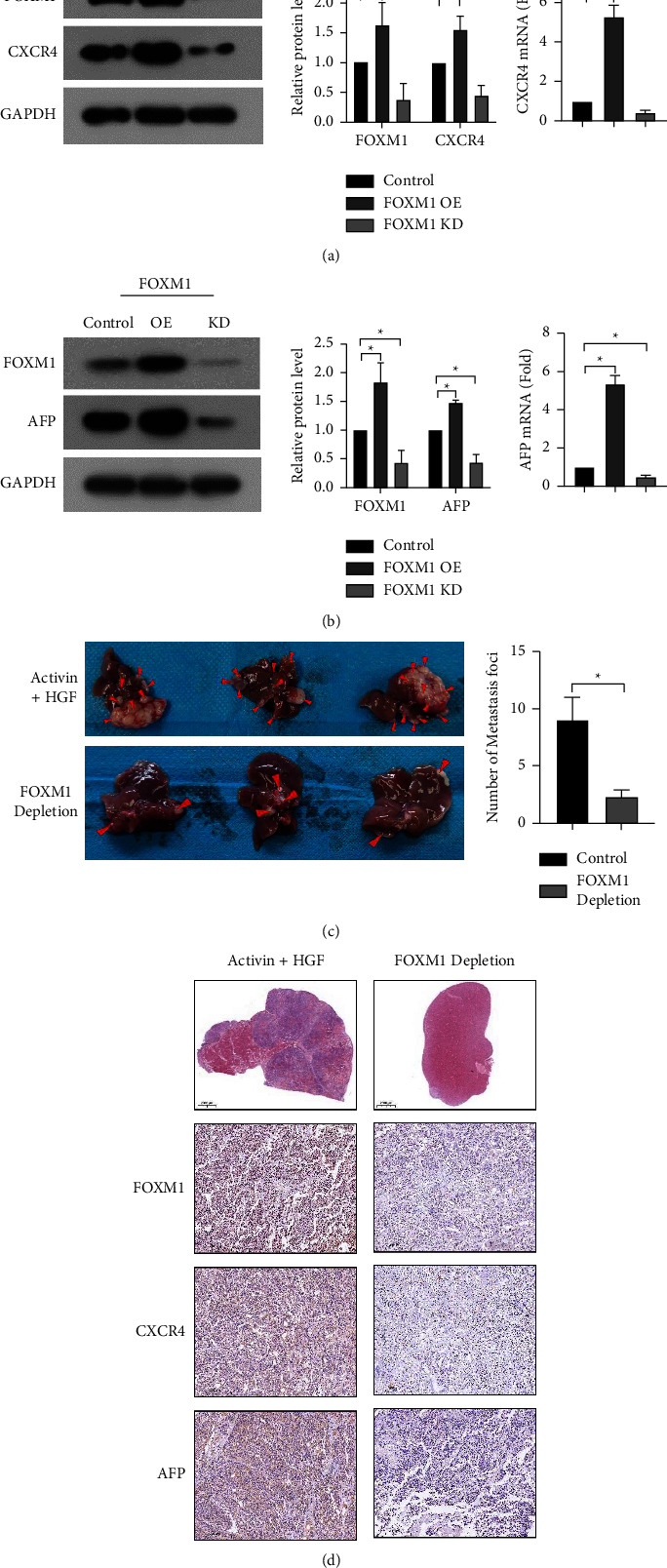
FOXM1 depletion attenuates the effects of sequential activin and HGF treatment on gene expression and CRC liver metastasis. (a-b) Western blot (left) and qPCR (right) analysis of CXCR4 and AFP expression in HCT116 cells with indicated treatments.  ^*∗*^*P* < 0.05*vs.* control. (c) WT or FOXM1-depleted HCT116 cells subjected to sequential treatment with activin and HGF were injected into the spleen of the nude mice. Metastatic lesions in the liver were counted.  ^*∗*^*P* < 0.05. (d) Immunohistochemical analysis shows that FOXM1 knockdown reduces the expression of AFP and CXCR and liver metastasis in nude mice.

Forkhead box M1 (FOXM1) is an important transcription factor that is overexpressed in many cancers [13–15]. It has been reported that FOXM1 is associated with cellular senescence [16] and stemness in CRC [17]. Next, we checked whether FOXM1 mediated the effect of activin and HGF on CRC cells. To address this, HCT116 cells were transfected with FOXM1 shRNA before sequential activin and HGF treatment. Transfection with FOXM1 shRNA resulted in significant downregulation of endogenous FOXM1 (Figure 4(a)). Both CXCR4 and AFP expression were decreased in FOXM1-depleted cells (Figures 4(a) and 4(b)). In vivo studies showed that FOXM1 depletion impaired the formation of liver metastases from HCT116 cells pretreated with activin and HGF. The average number of metastatic foci in the FOXM1 depletion and control groups was 9 ± 2 and 2.3 ± 0.58, respectively (Figure 4(c)). Immunohistochemical analysis indicated the downregulation of CXCR4 and AFP in FOXM1-depleted tumors (Figure 4(d)). Collectively, these data suggest that FOXM1 is required for CRC liver metastasis induced by activin and HGF.

### 3.4. Activin and HGF Induces the Expression and Phosphorylation of FOXM1 to Upregulate CXCR4 and AFP

**Figure 5:**
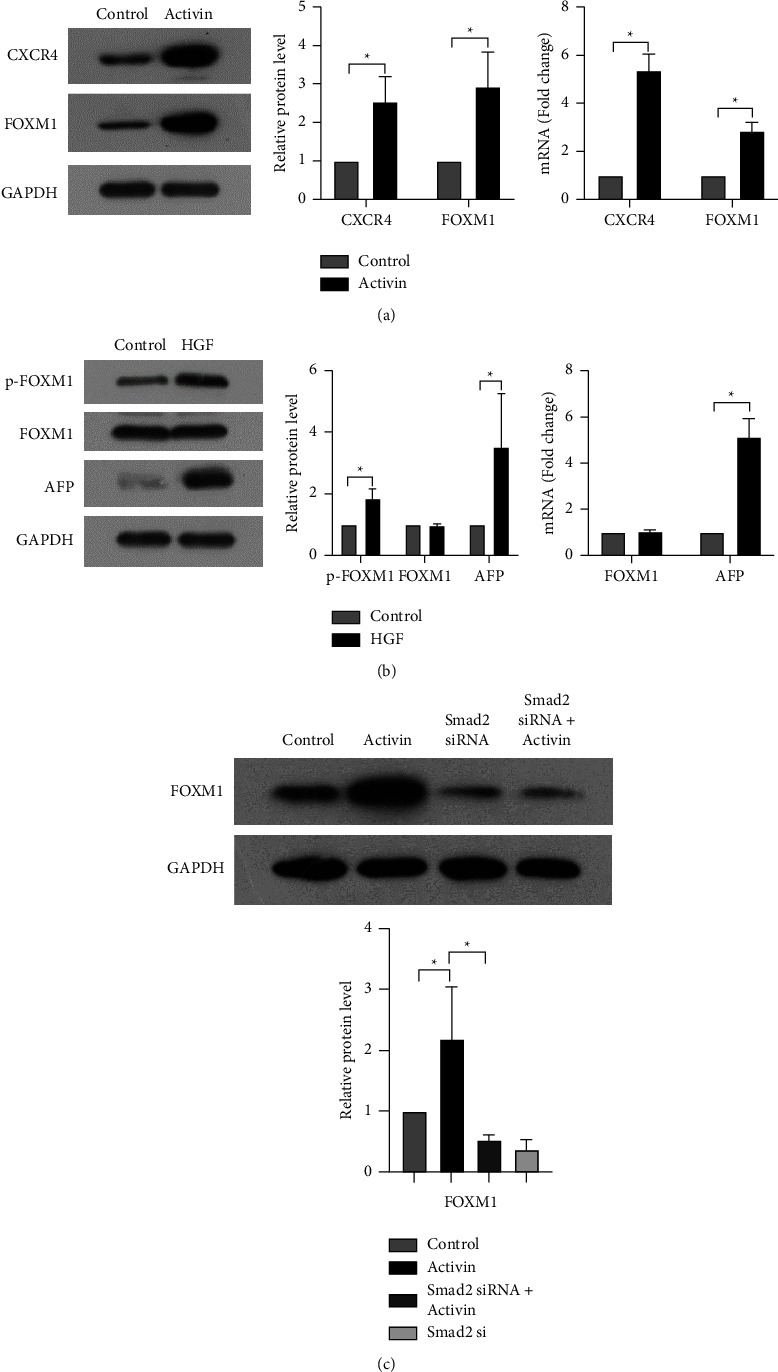
Activin and HGF induce the expression and phosphorylation of FOXM1 to upregulate CXCR4 and AFP through SMAD2. (a) Western blot and qPCR analysis of CXCR4 and FOXM1 expression in HCT116 cells induced by activin.  ^*∗*^*P* < 0.05*vs.* control. (b) Analysis of AFP, FOXM1, and phosphorylated FOXM1 (p-FOXM1) expression in HCT116 cells induced by HGF.  ^*∗*^*P* < 0.05*vs.* control. (c) Western blot analysis of FOXM1 expression in HCT116 cells treated with activin alone or together with SMAD2 siRNA.

Treatment with activin alone increased the mRNA and protein expression of FOXM1 in HCT116 cells (Figure 5(a)). In contrast, the expression of FOXM1 remained unchanged in response to HGF alone (Figure 5(b)). Although HGF treatment did not alter the total level of FOXM1, the phosphorylation of FOXM1 was enhanced by HGF treatment (Figure 5(b)). Given the stimulation of FOXM1 expression by activin and HGF, we asked whether FOXM1 could modulate the expression of CXCR4 and AFP. Notably, FOXM1 overexpression increased CXCR4 and AFP protein and mRNA levels in HCT116 cells, whereas FOXM1 knockdown reduced CXCR4 and AFP expression (Figures 4(a) and 4(b)). These results suggest that FOXM1 is involved in the upregulation of CXCR4 and AFP by activin activity and HGF.

### 3.5. Activin Induces FOXM1 Expression through SMAD2

It has been reported that activin can bind to SMAD2 [18]. We speculated that activin might increase FOXM1 expression through SMAD2. Interestingly, the knockdown of SMAD2 abrogated the upregulation of FOXM1 by SMAD2 (Figure 5(c)), indicating the importance of SMAD2 in modulating FOXM1 expression.

### 3.6. FOXM1 Directly Binds to the Promoter of *CXCR4*

**Figure 6:**
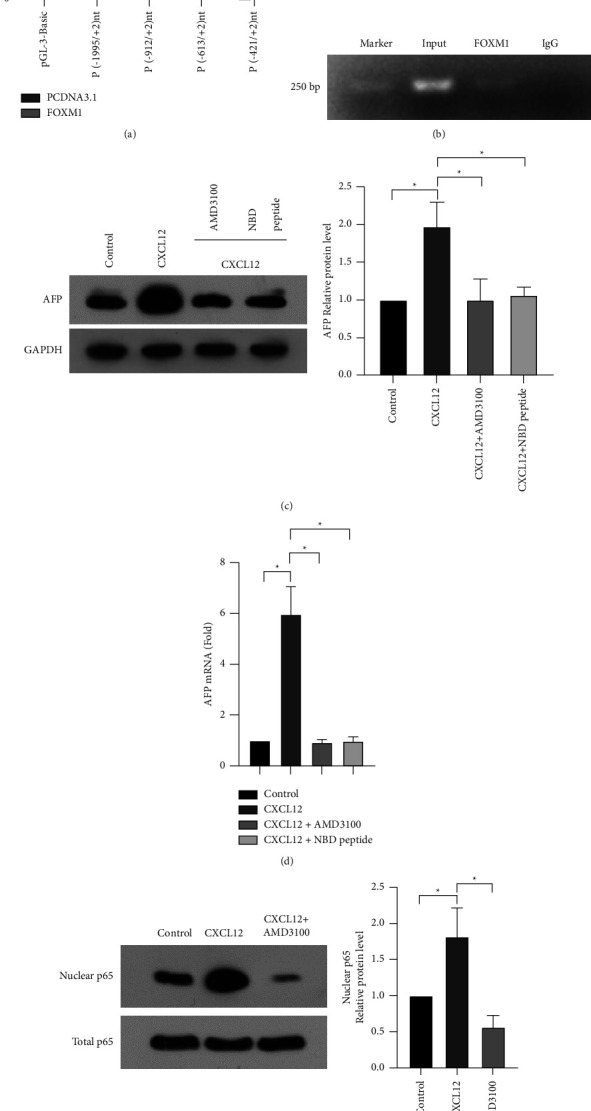
FOXM1 binds to the promoter of *CXCR4*, and CXCR4 regulates AFP expression through the NF-*κ*B pathway in CRC cells. (a) Luciferase reporter assays performed using the reporter constructs harboring different lengths of *CXCR4* promoter fragments.  ^*∗*^*P* < 0.05. (b) ChIP assays demonstrate that FOXM1 is enriched in the promoter of *CXCR4*. (c-d) Western blot and qPCR analysis of the effect of CXCR4 ligand CXCL12, CXCR4 inhibitor AMD3100, or NF-*κ*B inhibitor NBD peptide on the expression of AFP.  ^*∗*^*P* < 0.05*vs.* control. (e) Western blot analysis of the effect of CXCR4 ligand CXCL12 or CXCR4 inhibitor AMD3100 + CXCL12 on the expression of nuclear p65.

Next, we asked whether FOXM1 could directly bind to the promoter of *CXCR4*. To address this, we constructed the *CXCR4* promoter reporter pGL3-CXCR4 (position −1999 to +2) and its serial 5′-end deletion mutants, including P1 (position −912 to +2), P2 (position −613 to +2), and P3 (position −421 to +2). The reporter activities of the pGL3-CXCR4, P1, P2, and P3 constructs were increased by FOXM1 overexpression (Figure 6(a)). To validate the direct binding of FOXM1 to the *CXCR4* promoter region, we conducted a chromatin immunoprecipitation ChIP assay in HCT116 cells using an anti-FOXM1 antibody. The result showed that FOXM1 could bind to the regions of the *CXCR4* promoter (Figure 6(b)).

### 3.7. CXCR4 Regulates AFP Expression through the NF-*κ*B Pathway in CRC Cells

Next, we checked whether CXCR4 could regulate the expression of AFP in CRC cells. Of note, treatment with the CXCR4 ligand CXCL12 significantly upregulated AFP expression in HCT116 cells (Figures 6(c) and 6(d)). NF-*κ*B is well-known as a downstream target of CXCR4 signaling [19, 20]. As expected, CXCL12 treatment enhanced nuclear NF-*κ*B (p65) levels (Figure 6(e)). The CXCR4 inhibitor AMD3100 blocked CXCL12-induced nuclear enrichment of NF-*κ*B (Figure 6(e)), confirming the induction of NF-*κ*B activation by CXCR4. Most importantly, when NF-*κ*B activity was inhibited using a specific inhibitor NBD peptide, CXCL12-induced AFP expression was completely blocked (Figures 6(c) and 6(d)). Taken together, CXCR4 triggers the upregulation of AFP through the activation of NF-*κ*B signaling.

## 4. Discussion

The precise mechanism of CRC liver metastasis has yet to be determined. CSCs play an important role in primary tumor initiation, development, and metastasis [21]. Many regulators of CSCs have been found to be involved in CRC liver metastasis [22–24]. For instance, Yao et al. [24] reported that FBXW11 promotes stem-cell-like properties and liver metastasis in CRC. Sequential treatment with activin and HGF has been shown to induce the generation of liver progenitor-like cells [8, 9]. Rodrigues et al. [25] reported that activin contributes to the dedifferentiation of lung carcinoma cells into cancer stem cells. Liu et al. [10] reported that activin is implicated in colorectal cancer stem cell self-renew and tumor progression. Similarly, HGF can enhance the stem cell-like potential of cancer cells [26]. In support of the link between cancer stemness and metastasis, our data show that the 2 stem cell regulators, activin and HGF, can promote CRC invasion and liver metastasis.

It has been reported that activin and HGF can induce the generation of liver progenitor cells from human embryonic stem cells, which is characterized by the expression of CXCR4 and AFP [6]. Interestingly, our data show that activin and HGF can also upregulate the expression of CXCR4 and AFP in CRC cells. Moreover, CXCR4 and AFP expression is increased in CRC liver metastases. These results suggest that the prometastatic effects of activin and HGF are associated with the induction of CXCR4 and AFP expression.

Transcriptome analysis identifies many genes that are deregulated by sequential activin and HGF treatment. FOXM1 ranks among the most differentially expressed genes. In this study, we focus on FOXM1 because it is involved in cancer stemness and metastasis [27–29]. Dysfunction of FOXM1 occurs in several malignant cancers and is highly correlated with poor prognosis and enhanced metastasis [13–15]. The FOXM1-regulatory network is a critical predictor of poor prognosis in 18,000 cancer cases across 39 human malignancies [30]. Overexpression of FOXM1 can promote cancer cell migration and invasion and establish a premetastatic niche [31]. Regarding CRC biology, FOXM1 expression levels correlate with cancer progression, lymph node, liver metastasis, and high TNM stages [31]. Moreover, FOXM1 has been associated with stem cell-likeself-renewal in CRC [32]. Several studies have revealed the regulation of FOXM1 by activin and the HGF pathway [33, 34]. Activin A reduces FOXM1 expression in rat hepatocytes [33]. FOXM1 binds to the *Met* gene promoter and enhances HGF/Met signaling in pancreatic ductal adenocarcinoma cells by transcriptionally increasing Met expression [34]. Our data show that the expression of FOXM1, similar to CXCR4 and AFP, is upregulated in liver metastases of CRC. Knockdown of FOXM1 reduces CXCR4 and AFP expression levels in HCT116 cells. Most importantly, depletion of FOXM1 blocks liver metastasis of CRC cells with activin and HGF treatment in nude mice. Taken together, our data validate that FOXM1 is essential for the prometastatic effect of activin and HGF on CRC cells.

FOXM1 is a ubiquitously expressed transcription factor and has the capacity to trigger the transcription of a large number of genes [15–17]. Protein phosphorylation is required for FOXM1 function [16]. Joshi et al. [35] reported that MELK-mediated FOXM1 phosphorylation plays an essential role in the proliferation of glioma stem cells. Our data show that activin alone increases the expression of FOXM1 in CRC cells. Moreover, HGF alone enhances the phosphorylation of FOXM1 without altering the total level of FOXM1. These results provide a rationale for the treatment of CRC cells with sequential activin and HGF.

Biochemical studies further demonstrate that SMAD2 is implicated in the activin-induced upregulation of FOXM1. AFP expression can be induced by CXCL12/CXCR4 signaling, which is blocked by an NF-*κ*B inhibitor. In glioblastoma multiforme cells, CXCL12/CXCR4 upregulates FOXM1 expression, promoting tumor migration and invasion [36]. Our results support that FOXM1 can directly increase CXCR4 expression in CRC cells by binding to the promoter of *CXCR4*. Hence, FOXM1 and CXCL12/CXCR4 may form a positive feedback loop in the CRCLM; however, this hypothesis requires further investigation. In rat hepatocytes, activin A strikingly downregulates FOXM1 expression. Our results demonstrate that activin activates and increases FOXM1 expression. The opposite effects of activin on FOXM1 induction may be attributed to the different selected cell lines, suggesting that the functions of activin and FOXM1 in mature hepatocytes radically differ from those in CRC cells. CXCR4 is a marker of normal and malignant stem cells, and CXCL12/CXCR4 signaling is a crucial regulator of stem cell trafficking and cancer cell metastasis [37]. CXCR4-overexpressing CRC cells have been shown to exhibit trafficking functions and metastasis-initiating capacity [38]. However, the detailed crosstalk pathway among activin, FOXM1, CXCR4, and NF-*κ*B should be examined.

In conclusion, our results indicate that sequential treatment with activin and HGF promotes CRC invasion and liver metastasis. FOXM1 mediates the prometastatic effects of activin and HGF on CRC, which involves the induction of CXCR4 and AFP. Our work provides novel therapeutic targets for inhibiting liver metastasis of CRC.

## Figures and Tables

**Table 1:** Primers used in this study.

	Forward	Reverse
*β*-actin	5′-CCACGAAACTACCTTCAACTCC-3′	5′-GTGATCTCCTTCTGCATCCTGT-3′
FOXM1	5′-GGAGGAAATGCCACACTTAGCG-3′	5′-TAGGACTTCTTGGGTCTTGGGGTG-3′
AFP	5′-TGCAGCCAAAGTGAAGAGGGAAGA-3′	5′-CATAGCGAGCAGCCCAAAGAAGAA-3′
CXCR4	5′-TCTGTGACCGCTTCTACC-3′	5′-AGGATGAGGATGACTGTGG-3′
CHIP CXCR4	5′-ATCCCGCTTCCCTCAAACTT-3′	5′-ACAAACTGAAGTTTCTGGCCG-3′

## Data Availability

All the data used to support the findings of this study are included within the article.
